# The role of mindfulness in improving quality of life among student-athletes: a pilot mediation study

**DOI:** 10.3389/fpsyg.2025.1479584

**Published:** 2025-04-24

**Authors:** Lis Johles, Peter Molander, Carolina Lundqvist

**Affiliations:** ^1^Department of Social and Psychological Studies, Karlstad University, Karlstad, Sweden; ^2^Department of Behavioural Sciences and Learning, Linköping University, Linköping, Sweden; ^3^Department of Health, Medicine, and Caring Sciences, Pain and Rehabilitation Centre, Linköping University, Linköping, Sweden; ^4^Athletics Research Center, Linköping University, Linköping, Sweden

**Keywords:** athletes, body scan, mediation analysis, relaxation, quality of life

## Abstract

**Introduction:**

There has been a growing interest in mindfulness research during the past three decades. However, studies investigating the mediating mechanisms of mindfulness on student-athletes and their quality of life (QoL) are sparse. The purpose of this pilot study was to examine if the effects of a brief body scan intervention on QoL among student-athletes would mediate a change in five facets of mindfulness (acting with awareness, describing, non-judgment, non-reactivity, and observing).

**Methods:**

Swedish student-athletes (*n* = 99; 40% female participants) were recruited from six provincial high schools specialized in sports (basketball, *k* = 5; cross-country skiing, *k* = 1). The student-athletes were randomized into two arms: a 4-week body scan (intervention group) and a 4-week relaxation (active control group). Participants completed self-reported measures of QoL and mindfulness three times: at baseline; at follow-up, 4 weeks after baseline; and 8 weeks after baseline. A simple mediation analysis was conducted.

**Results:**

Results showed no significant differences between the body scan and relaxation on QoL change (c-path). There were no significant associations between the independent variable (body scan and relaxation) and the mediator Five Facet Mindfulness Questionnaire (FFMQ) (a-path). Results did not demonstrate any significant indirect associations between FFMQ and QoL (b-path) for the five facets of FFMQ. In conclusion, body scans did not have any effect on student-athletes’ QoL.

**Discussion:**

This study provides a first step toward investigating facets of mindfulness and QoL among student-athletes. No direct associations were revealed in this pilot study. Future research should refine mindfulness interventions and explore diverse mindfulness practices to better understand which facets of mindfulness may be helpful for student-athletes’ QoL.

## 1 Introduction

Research indicates that mindfulness is associated with multiple positive outcomes, including improved mental health and reduced stress (Anderson et al., 2023; Baltzell, 2016; Bühlmayer et al., 2017; Keng et al., 2011; Shannon et al., 2020). In recent years, sports organizations and researchers have increasingly focused on athletes’ mental health (Küttel and Larsen, 2020; Lundqvist et al., 2024; Reardon et al., 2019; Zhang et al., 2021). For instance, the International Olympic Committee (2021) has underscored the importance of regarding mental health as equally significant as physical health for athletes (International Olympic Committee, 2021). The concept of mental health often encompasses both negative factors (e.g., stress and mental disorders) and positive ones (e.g., quality of life, QoL) (Delfin et al., 2022; Lundqvist et al., 2023; Lundqvist et al., 2024). According to WHOQOL Group (1995, p. 1403), QoL is defined as “an individual’s perception of their position in life in the context of the culture and value systems in which they live, and in relation to their goals, expectations, standards and concerns.”

Students who engage in intensive athletic activities with rigorous training schedules while pursuing academic goals often face both sports-related and academic demands. These demands can heighten experiences of stress and overload, making it more difficult to maintain QoL (Davis et al., 2019; Thompson et al., 2022). Like many young adults in the general population, student-athletes are at risk of developing mental health issues, such as depression, anxiety, and social isolation (Lundqvist et al., 2023; Reardon et al., 2019). Research indicates that stigma surrounding mental health support among athletes may discourage help-seeking, potentially exacerbating these issues (Gulliver et al., 2012). When considering the above circumstances, it is of great importance to teach athletes how to take care of their mental health and recovery. One possible avenue to promote athletes’ mental health and QoL could be through mindfulness. Mindfulness training has been shown to be helpful across studies among adolescents (Black, 2015).

According to Kabat-Zinn (2009, p. 4), mindfulness is defined as “paying attention in a particular way, on purpose, in the present moment, and non-judgmentally to the unfolding of experience moment by moment.” Core components of mindfulness, such as openness, curiosity, and acceptance of present internal reactions, can be valuable in athletic contexts (Bishop et al., 2004; Levin et al., 2015). Mindfulness-based interventions and programs are frequently utilized in sports psychology to help athletes develop an observing and accepting relationship with their thoughts, emotions, and bodily sensations (Gardner and Moore, 2017; Gross et al., 2018; Noetel et al., 2019; Petterson and Olson, 2017). The goal is to develop attention and awareness so that athletes can deliberately determine which internal experiences merit a response (Baltzell, 2016). There is growing evidence supporting the benefits of mindfulness for athletes (Birrer et al., 2012; Foster and Chow, 2020). For example, a recent meta-analysis by Wang et al. (2023) showed that mindfulness interventions significantly enhance athletic performance and mindfulness-related psychological outcomes, but the results were non-significant for mental health outcomes. Consequently, questions remain regarding the effectiveness of mindfulness practice in enhancing QoL (Birrer et al., 2012, 2023) and specific facets of mindfulness that may impact student-athletes’ QoL.

Body scan is a common mindfulness practice in which attention is systematically directed to various parts of the body, potentially increasing awareness of internal sensations and thereby aiding in stress management (Finucane and Mercer, 2006; Gan et al., 2022; Ussher et al., 2014). A previous study (Anand et al., 2021) demonstrated a significant positive correlation between QoL in adolescents via mindfulness. In addition, the relationship of gratitude with physical health and environmental QoL was fully mediated by mindfulness (Anand et al., 2021). Findings regarding body scan in elite athletic contexts are pointing in divergent directions. For instance, one brief body scan intervention produced no positive effect on heart rate among female basketball players (Aras et al., 2023), whereas another study combining body scan with additional mindfulness exercises reported improved QoL among athletes (di Fronso et al., 2022). These contradictory results highlight the need for more research examining whether body scan interventions might benefit athletes.

Unlike breathing exercises, which primarily direct attention to respiration, a body scan involves intentionally focusing on inner sensations throughout the mind and body. The overarching aim of this pilot study is to examine if a brief body scan intervention can affect student-athletes’ QoL and to clarify whether changes in five mindfulness facets mediate this relationship. Relaxation exercises are used as an active control condition. Earlier research has highlighted divergent outcomes when comparing the effects of mindfulness with relaxation in athletes (Rooks et al., 2017). To better evaluate the efficacy of mindfulness practices for athletes’ QoL, further research is merited to determine the relative efficacy of mindfulness, relaxation training, and the effect on QoL. By analyzing potential mediating effects, we aim to identify the underlying relationships between body scan and the outcome QoL via facets of mindfulness. Such knowledge may be of interest when designing and refining interventions that effectively could support student-athletes’ overall QoL.

Our primary hypothesis is that student-athletes engaging in body scan intervention will benefit more on their QoL than the active control group with relaxation. Second, the intervention group practicing body scan is expected to demonstrate an increase in all mindfulness facets compared to the active control group performing relaxation exercises. Third, improvements in mindfulness facets will mediate the relationship between the body scan intervention and scores of QoL.

## 2 Materials and methods

### 2.1 Study design

The secondary analyses described herein are part of a larger project (Johles et al., 2023). The interventions were conducted in high school settings in Sweden. Participants were randomly assigned to intervention or control groups using block randomization and distributed into the two groups.

### 2.2 Participants

**Figure 1:**
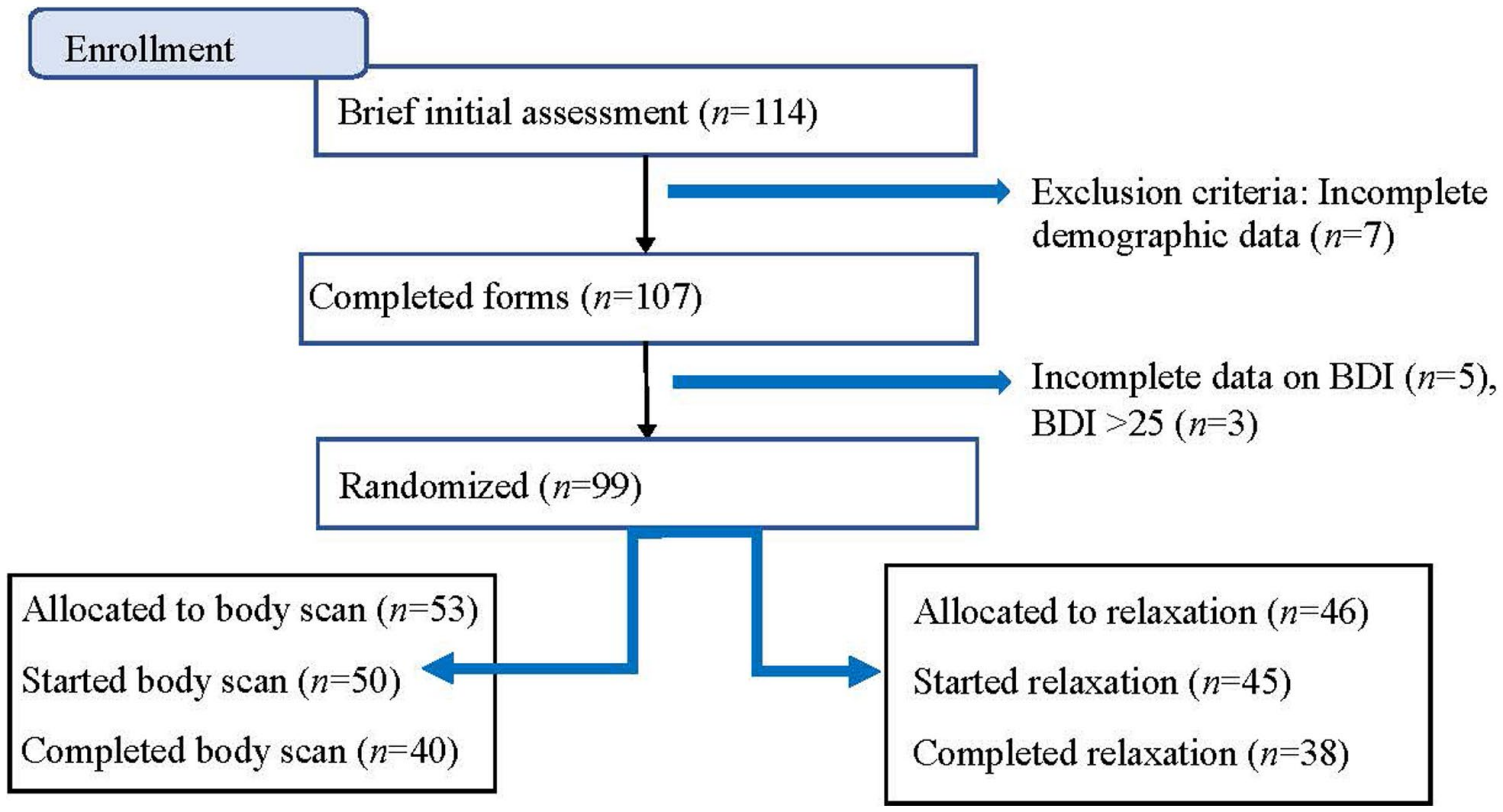
Flowchart.

The inclusion criteria were as follows: (a) competitive student-athletes and semi-elite who were active in their sport at the time of this investigation; and (b) could understand, write, and read Swedish. Competitive and semi-elite athletes were defined according to Swann et al. (2015). In other words, competitive athletes were regularly competing at the highest level in their sport while semi-elite were competing below the top standard in their sport in Sweden (e.g., in talent programs and development programs). Participants had to be at least 15 years old. Student-athletes attending these schools follow a regular high school program combined with their elite sports program. Seven high schools specializing in programs for either cross-country skiers or basketball, enrolled in the Swedish National Sports program (NIU), were approached. Schools were located all over Sweden and in both urban and rural regions. A total of 114 participants were recruited through coaches and teachers. Exclusion criteria were students who scored above cutoff (i.e., ≥26 points) on the Beck’s Depression Inventory (BDI-I; Beck et al., 1988; Beck and Steer, 1996), injured students who were unable to practice their sport, and students who could not understand, write, and read Swedish. Three student-athletes (*n* = 3) that scored above cutoff (i.e., ≥26 points) on Beck’s Depression Inventory (BDI-I; Beck et al., 1988; Beck and Steer, 1996) were excluded and recommended to seek help at the healthcare center at their school. Additionally, there was incomplete data on BDI-I for five participants (*n* = 5) and incomplete demographic information for seven participants (*n* = 7), whereby a total of 12 participants (*n* = 12) were excluded. The remaining participants (total: *n* = 99: women: *n* = 40; men: *n* = 59) were randomized into one of the two arms (see Figure 1 flow chart) and under the heading procedure. After data collection, all personal identifiers of participants (e.g., names, student IDs, or any other identifying information) were removed from the dataset. Each participant was assigned a unique alphanumeric code, which was used throughout the study to link data to individuals while maintaining confidentiality.

Table 1 shows baseline characteristics for included participants by intervention. The number of participants was equal after randomization. However, differences in the number of participants in each intervention group arose due to several reasons for exclusion (see flowchart Figure 1). The proportions of female participants and the type of sports (basketball vs. cross-country skiing) did not differ between categories of intervention.

**Table 1:** Demographic description of the participants at baseline by intervention category (*N* = 99 participants).

Characteristic	4 W body scan (*n* = 53)	4 W relaxation (*n* = 46)
Age, mean (SD)	16.8 (0.9)	17.0 (0.8)
Females, *n* (%)	21 (40)	19 (41)
Basketball, *n* (%)	44 (83)	36 (78)
Years in current club	4.2 (4.0)	4.2 (2.5)
Hours of practicing the sport per week	12.9 (3.6)	13.3 (2.8)
Performance (years), mean (SD)	8.8 (2.5)	9.2 (2.5)
Years competing at elite level, mean (SD)	2.8 (2.2)	2.7 (1.3)

Data presented as mean (SD) unless otherwise indicated. SD, standard deviation.

### 2.3 Measures

Measures of mindfulness and QoL were collected at baseline (T0), after 4 weeks (T1), 8 weeks (T2), and 16 weeks (T3) after baseline. At baseline, we also assessed depressive symptoms to identify and exclude student-athletes with severe depression. The completed measures were reported by each participant and collected by the first author or the research assistants visiting each school.

#### 2.3.1 The Beck depression inventory (BDI–I)

The Swedish version of the revised 21-item BDI (BDI-I; Beck et al., 1988; Beck and Steer, 1996) was used to screen for indications of severe depression. BDI-I has adequate properties for screening and is also free of charge. The BDI has been validated in multiple cultures and languages, increasing its applicability in diverse populations (Hammond et al., 2013; Wang and Gorenstein, 2013). The BDI-I assesses cognitive and somatic depression, and each item is rated according to the past 2 weeks on a four-point Likert scale from 0 to 3. The BDI-I can thereby range between 0 and 63. The cutoff levels are 0–14 (no depression), 14–20 (mild depression), 20–26 (moderate depression), and above 26 (severe depression). The internal consistency and 1-week test–retest reliability have been shown as acceptable in clinical and non-clinical samples (Beck et al., 1988; Beck and Steer, 1996). The internal consistency in this pilot study was 0.87. BDI-I was only used for the exclusion of participants with indications of clinical levels of depression.

#### 2.3.2 The Brunnsviken brief quality of life scale (BBQ)

The Swedish version of BBQ (Lindner et al., 2016) covers six areas (i.e., leisure time, view on life, creativity, learning, friends and friendship, and view of self), empirically shown to be important for a single QoL construct. Examples of questions include “My leisure time is important for my QoL,” “Being able to be creative is important for my QoL,” and “I am satisfied with myself as a person. I like and respect myself.” Participants rated both the level of importance and their degree of satisfaction with each life area. The ratings of importance and degree of satisfaction with each are multiplied, and the products are summed. The item response format ranges from 0 (do not agree at all) to 4 (agree completely), generating an *α* total score between 0 and 96. A higher score indicates a higher QoL. The BBQ has demonstrated satisfactory reliability, with good concurrent and convergent validity (Lindner et al., 2016). In our pilot study, Cronbach’s alpha was 0.76 for the total scale (subscales: leisure time = 0.89, view on life = 0.86, creativity = 0.84, learning = 0.87, friends/friendship = 0.76, and view of self = 0.87).

#### 2.3.3 The five facet mindfulness questionnaire (FFMQ)

The FFMQ (Baer et al., 2008, 2019) is a self-report measure that includes 39 items that measure five facets of mindfulness. The facets are observing (i.e., a way to notice feelings, sensations, and thoughts, one example of this item is: When I am having a shower or a bath, I am paying attention to the sensation of water against my body), describing (i.e., the ability to express and verbalize feelings and thoughts, one example of the items is; “I can easily find words to describe my feelings”), acting with awareness (i.e., non-distractive, not doing things on auto-pilot, focusing on the present behavior, one example of this items is, “I have difficulties paying attention in the present moment”), non-judgment (i.e., self-acceptance and tolerance of one’s feelings and thoughts, one example of this item is; “I am judging my feelings to be either good or bad”), and non-reactivity (i.e., ability to step back from thoughts and feelings without having to act on inner reactions, one example of this item is; “I notice my feelings without having to react on them”). All items were rated on a five-point Likert scale ranging from 1 (never or very rarely true) to 5 (very often or always true). Each facet can be calculated separately, which was done in this investigation. A high total score on facets demonstrates an elevated level of mindfulness (Baer et al., 2008). In this pilot study, mindfulness was measured using the Swedish-validated version of FFMQ (29 items), which has shown satisfactory reliability and factor validity (Lilja et al., 2011, 2020). The Swedish version of FFMQ was used since no other mindfulness scales have been validated in Sweden. Cronbach’s alpha for facet in FFMQ in this study ranged from 0.66 to 0.84 (acting with awareness = 0.77, describing = 0.83, non-judgment = 0.84, non-reactivity = 0.66, and observing = 0.71). The respondent age for the Swedish version of FFQM is 16 years of age. When the revised Swedish version of FFMQ was validated (Lilja et al., 2011, 2020), a population (*n* = 73) between 16 and 21 years of age (not athletes) was included, which is a comparable age group with our population.

### 2.4 Procedure and intervention

After ethical approval from the Regional Ethics Board, recruitment started in September 2016 and continued during the fall of 2016. All schools were contacted via e-mail and received a letter explaining the purpose of the pilot study. A coach or a teacher informed their student-athletes that a researcher had invited them to a meeting where more detailed information would be given. The first author visited the schools and explained the purpose of the investigation, and all student-athletes had the opportunity to ask questions. According to Swedish law, no consent from parents or guardians is required from 15 years and older for participating in research that does not include treatment. Interested student-athletes provided written informed consent to participate in the pilot study and subsequently filled in the information on their age, sex, years in current club, months injured, years of athletic experience, and years competing (see Table 1: demographic description of the participants at baseline). The BDI, FFMQ, and BBQ were administrated in random order. The questionnaires were completed in a classroom or an auditorium, and the first author or a research assistant was present to answer any questions. Lists of anonymous identification numbers were used to randomly allocate participants to one of the two arms. Participants completed baseline measurements (T0) before block randomization was performed. After data collection, all personal identifiers of participants (e.g., names or any other identifying information) were removed from the dataset. Each participant was assigned a unique alphanumeric code, which was used throughout the study to link data to individuals while maintaining confidentiality. The codes were written down randomly, followed by dividing the codes by five participants for each high school class into one of the two arms. Within each school and class, student-athletes were randomly assigned to two arms: 4-week body scan intervention or 4-week relaxation as active control groups. Participants were individually informed about their allocation through a written announcement at their schools, including their individual code, just before the start of the intervention. Teachers, coaches, and classmates did not receive any information about each participant’s allocation.

The active intervention groups took part in the 8-min audio-guided body scan exercise recorded by the first author. The practice of relaxation was recorded by the first author in 2002. Both recordings were the same length on the audio. The body scan practice was recorded in September 2016 and inspired by Kabat-Zinn (2005). The participants received the audio-guided exercises via e-mail and were asked to do the practice regularly on their own without any support from coaches or teachers (see Appendix for a translated transcript of the exercise). Before and after listening to the audio recording, the participants were asked to self-evaluate their frequency of practice, including the perceived level of being in the present moment (level 0–100, where 0 is not present in the moment and 100 = maximum present in the moment). However, due to lower response rate, these self-evaluated compliance measures were left out of the outcomes. Only eight participants returned their compliance measures, and compliance measures were not correctly filled in. Instructions in the body scan practice included, for example: “Just pay attention to what you become aware of.” What do you notice? “Just notice the contact with your left foot against the floor.” The participants were encouraged to practice the body scan regularly; however, the frequency of the practice was not decided prior to the onset of the intervention. All participants were asked about their previous experience with body scan or relaxation exercises. While a few participants had tried relaxation, none had previously used body scan. No prior experience with body scan was necessary to follow the instructions.

The participants in the active control group with relaxation received an 8-min audio-guided relaxation exercise inspired by Öst’s brief applied relaxation (Öst, 1987) and recorded by the first author (Johles, 2002; Johles et al., 2023). Detailed information about the relaxation practice is available in the study by Johles et al. (2023).

## 3 Data analysis

Descriptive statistics and Cronbach’s alpha were calculated using IBM Corp (2017). Simple mediational analyses (separate models were run on each outcome) were conducted using random slopes extracted from separately run mixed effects models. Effect sizes were based on the mediation proportion (PM), which is the ratio of the indirect effect to the total effect (Hayes, 2018; MacKinnon, 2008). In line with the procedures by Carrión et al. (2017) and Nock (2007), we tested the products of the (1) independent variable (groups) to the mediator change from baseline to post-treatment (T0-T2), (a-path) and (2) the mediator to the dependent variable, which is the change in QoL outcomes at baseline to follow-up when the independent variable is considered (b-path). Random slopes (i.e., individual change) in mindfulness (FFMQ facets) served as mediators and QoL (BBQ) as the outcome. To examine the potential mediating role of FFMQ in predicting changes in QoL, a series of linear mixed-effects models with random intercepts were estimated using the lme4 and lmerTest packages in R (Bates et al., 2015; Kuznetsova et al., 2017). Each potential mediator was tested in a separate model to assess its individual association with changes in QoL.

## 4 Results

Overall, the intervention with the body scan did not increase levels of QoL more than relaxation, as described in Table 2. The results showed no significant interaction effect between group and time [b = 0.29, SE = 0.96, *t*(161.39) = 0.30, *p* = 0.76]. There was no significant main effect of group [b = −0.91, SE = 1.13, *t*(151.83) = −0.81, *p* = 0.42] or time [b = 0.74, SE = 0.70, *t*(162.57) = 1.06, *p* = 0.29].

**Table 2:** Estimated marginal means by time point and group.

Time point	Group	M	SE	95% CI
1	4RX	37.1	1.22	[35.5, 38.7]
1	4BS	36.1	1.19	[34.6, 37.7]
2	4RX	37.3	1.26	[35.6, 39.0]
2	4BS	36.7	1.23	[35.0, 38.3]
3	4RX	37.8	1.26	[36.1, 39.5]
3	4BS	37.2	1.23	[35.5, 38.8]
4	4RX	35.1	1.14	[32.8, 37.5]
4	4BS	33.7	1.29	[31.0, 36.4]

RX, Relaxation; BS, Body scan; M, Mean; SE, Standard Error; CI, Confidence interval.

As displayed in Table 2, no significant associations were found at the 16-week follow-up, indicating that the variables of interest did not show meaningful relationships at this time point, since the 16-week data did not contribute significant associations. Therefore, only data from the 4-week assessment and the 8-week follow-up were used for the mediation analysis to maintain relevant statistical interpretability.

### 4.1 Mediation analyses

The results of the mediation analyses are presented in Tables 3–5. Body scan and relaxation were entered as group variables, and slopes from five facets (FFMQ) were included as separate mediators analyzed one at a time. QoL slopes were entered as the dependent variable. We hypothesized that all potential facets of mindfulness in FFMQ would change to a greater extent for those who received body scan in comparison to relaxation training, but this hypothesis could be rejected. As shown in Table 4, there were no significant associations between the independent variable (body scan and relaxation (= active control group)) and the mediator FFMQ (a-path). There was no significant relationship between the mediator FFMQ and the dependent variable QoL (b-path) (see Table 4).

**Table 3:** Path coefficients for individual mediators.

Mediator	a-path estimate	a-path *p*-value
Non-react	0.15	0.17
Observe	0.10	0.21
Act with awareness	−0.17	0.16
Describing	0.03	0.74
Non-judge	0.01	0.95

Estimates and *p*-values correspond to the a-path of each mediator.

**Table 4:** Path coefficients for model with all mediators.

Mediator	b-path estimate	b-path *p*-value
Non-react	0.14	0.28
Observe	0.09	0.37
Act with awareness	−0.27	0.08
Describing	−0.10	0.40
Non-judge	0.13	0.32

Estimates and *p*-values correspond to the b-path of each mediator.

**Table 5:** Direct and total effects.

Effect	Estimate	*p*-value
c-path (direct)	1.23	0.76
Total effect (c)	1.28	0.03

The direct effect (c-path) and total effect estimates are reported.

Consequently, the mediator on the dependent variable QoL (c-path) was not linked to any of the interventions (Table 5), and the a-path was not significant for any of the five facets, as shown in Table 3. Brought together, the intervention with body scan did not influence the facets of FFMQ.

## 5 Discussion

This pilot study investigated the mediation effects of a 4-week body scan intervention compared to a 4-week relaxation on mindfulness and QoL among student-athletes, with a particular focus on the five facets of mindfulness (FFMQ). The findings revealed no significant direct effects of either the body scan or relaxation interventions on QoL. This outcome contradicts prior research suggesting that mindfulness training improves QoL in student-athletes (Jones et al., 2020), although, as previously mentioned, earlier research has demonstrated mixed outcomes linked to body scan. These results from our study highlight the complexity of understanding the specific mechanisms through which mindfulness interventions affect QoL. Path analyses demonstrated that the interventions did not directly influence QoL. The absence of significant relationships between the interventions and the mediator facets of mindfulness (a-path) raises questions about the importance of specific mindfulness components in mediating QoL among student-athletes. The lack of QoL effects may stem from the difficulties with collecting and measuring compliance measurements. In other words, since few measurements were submitted, it is difficult to know how many times participants applied the exercises. The body scan’s similar effectiveness to relaxation suggests that it is relaxational component plays a key role. Both practices emphasize present-moment awareness and breath focus, though relaxation involves deliberate muscle tension release and controlled breathing, while mindfulness observes the breath naturally. These shared elements may explain the lack of differences between groups (Feldman et al., 2010).

The left-out positive association of “acting with awareness” and “non-reactivity” contradicts prior research indicating that dispositional mindfulness can buffer against depression among college athletes (Glass et al., 2019). Similarly, the left-out significant role of “non-reactivity” challenges earlier findings linking mindfulness and global wellbeing (Foster and Chow, 2020). In contrast to our expectations, the “observing” facet did not show any significant relationship with QoL, which contradicts prior findings linking high levels of “observing” to mindfulness benefits (Lilja et al., 2013). This discrepancy might stem from the cognitive and emotional characteristics of the athletic sample in our study. For example, athletes may already excel at perceiving and recognizing stimuli, rendering this facet less impactful within the intervention context.

Several other factors may explain the results. The athletes in this pilot study were likely to have well-developed skills in “acting with awareness,” which is critical in sports training and competition. Additionally, age, gender, and other recovery practices may have influenced QoL outcomes indirectly. The lack of significant differences between body scan and relaxation interventions suggests overlapping components, such as awareness of the body and breathing, which may have diluted potential effects (Birrer et al., 2023; Chiesa and Malinowski, 2011). Body scan emphasizes accepting and observing the breath, whereas relaxation focuses on reducing physical arousal and controlled breathing (Feldman et al., 2010; Feruglio et al., 2021). In conclusion, this study suggests that mindfulness with a brief body scan practice is not an effective intervention.

### 5.1 Strengths, limitations, and future pathways

To the best of our knowledge, this is the first longitudinal mediation analysis exploring the effects of a brief body scan intervention on QoL among Swedish student-athletes aged 16–19 years. A strength of this pilot study is its randomized controlled trial (RCT) design and high ecological validity, achieved through collaboration with coaches and teachers in scholastic athletic settings. Another strength is the inclusion of an active control group. Key outcome measures, such as QoL and mindfulness, were assessed individually to minimize the potential confounding effects of peer influence. However, all participants completed the measures in a classroom setting, which may have introduced social and emotional pressures during the assessment. There may have existed variability in adherence to the interventions, including multitasking during audio recordings, which was difficult to control and may have influenced outcomes. We made an attempt to measure experienced levels of relaxation and experienced presence at the moment directly after the applied practice. However, due to lower response rate, this was left out of the outcomes. In the context of this study, the relative anonymity among participants is advantageous for maintaining the objectivity of the intervention effects. Nonetheless, we acknowledge that future studies could explore the influence of pre-existing relationships on intervention outcomes in a more controlled manner, as this may offer valuable insights into the social dynamics of group-based interventions. Furthermore, participants may have discussed their progress and training experiences in positive terms, creating an atmosphere of motivation and encouragement. Such group dynamics could enhance participants’ engagement with the intervention and influence the outcomes. Considering the aforementioned challenges and the fact that many previous investigations have used small samples and have methodological limitations, it still remains unclear whether body scans are sufficient to enhance athletes’ QoL.

Future studies should address these limitations by broadening the range of sports and age groups and examining additional mindfulness practices (e.g., extended body scans, yoga, and walking meditation) and/or passive control groups. Investigating the interplay between mindfulness facets and emotion-regulation processes may further elucidate mindfulness as a mechanism of change for athlete interventions (Bird et al., 2021; Birrer et al., 2023).

## 6 Conclusion

In conclusion, neither body scan nor relaxation demonstrated significant direct effects on QoL among student-athletes. No observed indirect associations of specific mindfulness facets on QoL were discovered. These findings underscore the need for future longitudinal research to refine the understanding of mindfulness interventions and clarify the impact of facets of mindfulness on QoL. Designing studies with passive control groups and including diverse mindfulness techniques could yield more robust insights into effective strategies for supporting student-athletes’ QoL.

## Data Availability

The raw data supporting the conclusions of this article will be made available by the authors, without undue reservation.
